# Tuberous Sclerosis Complex cell‐derived EVs have an altered protein cargo capable of regulating their microenvironment and have potential as disease biomarkers

**DOI:** 10.1002/jev2.12336

**Published:** 2023-06-19

**Authors:** Muireann Ní Bhaoighill, Juan M. Falcón‐Pérez, Félix Royo, Andrew R. Tee, Jason P. Webber, Elaine A. Dunlop

**Affiliations:** ^1^ Tissue Microenvironment Group School of Medicine Cardiff University Cardiff UK; ^2^ Division of Cancer and Genetics School of Medicine Cardiff University Cardiff UK; ^3^ Exosomes Lab. CICbioGUNE‐BRTA Parque Tecnologico Derio Spain; ^4^ Centro de Investigación Biomédica en Red de enfermedades hepáticas y digestivas (CIBERehd) Madrid Spain; ^5^ IKERBASQUE Basque Foundation for Science Bilbao Spain; ^6^ Institute of Life Science Swansea University Medical School Swansea University Swansea UK

**Keywords:** extracellular vesicles (EVs), fibroblasts, mTORC1, TSC2, tuberous sclerosis complex (TSC), tumour microenvironment

## Abstract

Hyperactivation of mechanistic target of rapamycin complex 1 (mTORC1) is a feature of many solid tumours and is a key pathogenic driver in the inherited condition Tuberous Sclerosis Complex (TSC). Modulation of the tumour microenvironment by extracellular vesicles (EVs) is known to facilitate the development of various cancers. The role of EVs in modulating the tumour microenvironment and their impact on the development of TSC tumours, however, remains unclear. This study, therefore, focuses on the poorly defined contribution of EVs to tumour growth in TSC. We characterised EVs secreted from *TSC2*‐deficient and *TSC2*‐expressing cells and identified a distinct protein cargo in *TSC2*‐deficient EVs, containing an enrichment of proteins thought to be involved in tumour‐supporting signalling pathways. We show EVs from *TSC2*‐deficient cells promote cell viability, proliferation and growth factor secretion from recipient fibroblasts within the tumour microenvironment. Rapalogs (mTORC1 inhibitors) are the current therapy for TSC tumours. Here, we demonstrate a previously unknown intercellular therapeutic effect of rapamycin in altering EV cargo and reducing capacity to promote cell proliferation in the tumour microenvironment. Furthermore, EV cargo proteins have the potential for clinical applications as TSC biomarkers, and we reveal three EV‐associated proteins that are elevated in plasma from TSC patients compared to healthy donor plasma.

## 1 INTRODUCTION

Mechanistic target of rapamycin complex 1 (mTORC1) is a principal regulator of cell growth and metabolism (Ben‐Sahra & Manning, 2017; Saxton & Sabatini, 2017). Intracellular signalling governed by mTORC1 is tightly controlled and well elucidated, with its activation dependent on the amino acid levels and the energy status of the cells. mTORC1 signalling controls a programme of anabolic processes that promotes protein synthesis and cell growth (Laplante & Sabatini, 2013). Dysregulation of mTORC1 signalling is an anabolic driver in many cancer types that contributes to uncontrolled cell growth (Hua et al., 2019). Dysregulated mTORC1 signalling is also well documented in the inherited condition Tuberous Sclerosis Complex (TSC), due to loss of function mutations in the upstream mTORC1 regulators, *TSC1* and *TSC2*. Given that mTORC1‐hyperactivity is a hallmark driver of TSC‐associated tumours, TSC preclinical models are commonly used to study mTORC1‐active tumour biology (Dodd & Dunlop, 2016).

It is not just the molecular characteristics of the tumour that influence its growth and response to treatment. Tumour cells are in continuous crosstalk with the surrounding non‐malignant tissue. This helps to cultivate a pro‐tumoral environment that supports the successful growth and survival of the tumour cells (Joyce & Pollard, 2008; Valkenburg et al., 2018). This tumour microenvironment (TME) comprises a range of cellular and non‐cellular components, including fibroblasts, inflammatory immune cells, tumour‐associated vasculature and extracellular matrix (Luga et al., 2012). There is evidence that these form a dynamic, heterogeneous niche that provides optimal conditions for tumour cells to grow and survive (Elia & Haigis, 2021) and can influence treatment efficacy (Hirata & Sahai, 2017). However, interactions between TSC cells and their TME remain unclear. Expanding our knowledge of TSC cell signalling to the TME could highlight previously unknown mechanisms of tumour growth and development, while also revealing potential targets for novel therapeutic strategies for both TSC and mTORC1‐driven cancers. Furthermore, inhibition of mTORC1 by rapalogs, the standard‐of‐care therapy for TSC‐associated renal angiomyolipoma (AML) and subependymal giant cell astrocytomas (SEGAs), has some therapeutic limitations and heterogenous efficacy (Bissler et al., 2008). Therefore, understanding how rapamycin‐treated tumour cells crosstalk with their TME is also beneficial to improve anti‐tumour treatment.

Intercellular communication can occur through a variety of means, but growing evidence points to the role of extracellular vesicles (EVs) as a means by which tumour cells communicate and modify the tumour microenvironment (Cho et al., 2012; Chowdhury et al., 2015; Webber et al., 2010). EVs are lipid membrane‐encapsulated vesicles that act as intercellular carriers of biomolecules, including RNA, protein and lipids. These biomolecules are packaged by the parent cell into the vesicles and can remain biologically functional when in contact with, or taken up by recipient cells (Maas et al., 2017).

In the cancer setting, tumour cell‐derived EVs have been shown to contribute to tumour development by driving the differentiation of stromal fibroblasts towards a pro‐angiogenic phenotype, capable of supporting tumour growth in vivo (Webber et al., 2015). Furthermore, it was recently shown that the RNA cargo of circulating EVs can be indicative of stromal cell activation and aggressive disease (Shephard et al., 2021). Both EV secretion rate and EV cargo can play a role in promoting these tumour‐supporting processes. Cancer cells are reported to secrete elevated amounts of EVs, compared to non‐malignant cells (Logozzi et al., 2009) and can deliver tumour‐derived bioactive molecules to recipient cells in the microenvironment and at distant sites (Peinado et al., 2012). Alterations in EV protein cargo could promote tumour‐supporting microenvironmental remodelling (Hoshino et al., 2015; Webber et al., 2016). Therefore, profiling EV protein cargo can be biologically insightful and provide mechanistic information on how EVs may optimise their TME for tumour growth and survival (Huang et al., 2021). This could reveal potential novel pathways for therapeutic intervention, or novel biomarkers for diagnosis and/or monitoring of disease (Dear et al., 2013; Keklikoglou et al., 2019; Raimondo et al., 2011; Simpson et al., 2012).

Current knowledge of EVs released by *TSC*‐deficient cells or mTORC1‐active cells, and their functional consequences within the TME, is limited. Recently, mouse cells lacking *Tsc1* or *Tsc2* proteins have been shown to secrete more EVs than their wild‐type counterparts (Kumar et al., 2021; Zadjali et al., 2020) and EVs from such cells can alter mTORC1 activity of wild‐type cells (Kumar et al., 2021; Patel et al., 2016). This has been attributed to the delivery of *Rheb* and *Notch* RNAs to wild‐type cells (Patel et al., 2016). However, little is known about the protein cargo of EVs from human *TSC*‐deficient cells, which could act more rapidly on target cells than RNA cargo and could also be more easily targeted therapeutically. To address this knowledge gap, we studied EVs released from *TSC2*‐expressing (*TSC2*+) and *TSC2*‐deficient (*TSC2*‐) cells derived from a TSC patient.

Following EV characterisation, we analysed their protein cargo and identified a distinct proteome profile within EVs secreted from *TSC2*‐ cells compared to that of EVs from *TSC2*+ cells. We show that EVs from *TSC2*‐ cells promote cell viability, proliferation and cytokine release from recipient wild‐type fibroblasts. Importantly, we show that rapamycin treatment can alter the EV protein cargo to resemble that observed in EVs from *TSC2*+ cells. Furthermore, EVs from rapamycin‐treated *TSC2*‐ cells had reduced capacity to stimulate cell proliferation in recipient fibroblasts compared to EVs from untreated *TSC2*‐ cells. These findings are previously unreported functions of rapalogs. There is a clinical need for simpler monitoring of TSC disease progression and treatment efficacy. In this study, we are the first to show the successful extraction of EVs from the blood of TSC patients. Three of our identified EV proteins were found to be elevated in TSC patient plasma, compared to healthy donor plasma, suggesting EVs as a potential source of novel biomarkers for TSC that can be obtained from patient blood.

## 2 MATERIALS AND METHODS

### 2.1 Cell culture

The patient‐derived angiomyolipoma cell line 621‐102 and the matched 621‐103 *TSC2* re‐expressing cell line were a kind gift from Prof Elizabeth Henske (Brigham and Women's Hospital, Boston, USA) and were derived as described in Hong et al. (2008). The cells were grown in Dulbecco's Modified Eagle's Media (DMEM), supplemented with 100 U/mL penicillin, 100 μg/mL streptomycin and 15% (v/v) fetal bovine serum (FBS) (all Sigma‐Aldrich, Gillingham, UK). *TSC2*‐deficent cells were treated where indicated with 10 ng/mL rapamycin (Merck, Gillingham, UK). Human pulmonary fibroblasts (HPF‐c) (Promocell, Heidelberg, Germany) were grown in low‐serum Fibroblast Growth Medium 2 (Promocell). All cell lines were cultured with FBS that had been depleted of EVs by ultracentrifugation at 100,000 × *g*, for 18 h, followed by serial filtration through 0.22 and 0.1 μm vacuum filters.

### 2.2 EV isolation from cell conditioned media

TSC cell lines were maintained at high‐density culture in CELLine adhere bioreactor flasks (Integra Biosciences Corp, Hudson, NH, USA) (Mitchell et al., 2008). Cell‐conditioned media was depleted of cells and cellular debris by centrifugation at 400 × *g* for 6 min, followed by centrifugation at 2000 × *g* for 15 min. EV isolation was performed by underlying cell‐conditioned media with a 30% (w/v) sucrose cushion based on the protocol described previously (Lamparski et al., 2002) prior to ultracentrifugation at 100,000*g* for 90 min and subsequent PBS wash and re‐pelleting of EVs by ultracentrifugation at 100,000 × *g* for 90 min. The resulting EV pellet was resuspended in PBS. EV protein concentration was quantified by Micro BCA™ Protein Assay Kit (Thermo Fisher Scientific) as per the manufacturer's protocol.

### 2.3 Cryo‐electron microscopy

Cryo‐electron microscopy (cryo‐EM) was conducted in the laboratory of Prof Juan Falcon‐Perez (CIC bioGUNE, Bilbao). Briefly, EV samples were fixed onto glow‐discharging holey carbon 200‐mesh copper grids Quantifoil Micro Tools GmbH, (Großlöbichau, Germany) and subjected to vitrification using a Vitrobot (Maastricht Instruments BV, Maastricht, The Netherlands). Imaging of vitrified samples was conducted at liquid nitrogen temperature using a JEM‐2200FS/CR transmission cryo‐electron microscope with a field emission gun, operated at an acceleration voltage of 200 kV. 33 TSC2+ and 46 TSC2‐ microscopic fields were examined per sample using ImageJ (version 1.50i).

### 2.4 Nanoparticle tracking analysis

Samples were diluted in particle‐free water (Fresenius Kabi, Runcorn, UK), as required, to obtain concentrations within the specified linear range of the instrument (up to 2 × 10^9^ particles per mL). Analysis was performed on a NanoSight™ NS300 system at 25°C with a 488 nm laser. Three videos of 2 min were recorded in light scatter mode under controlled fluid flow (pump speed 80). Videos were analysed by batch analysis using the NTA 3.1 software (version 3.1 build 3.1.54), where minimum particle size, track length and blur were set at ‘automatic’. The area under the histogram for each triplicate measurement was averaged and used as a particle concentration measurement. Nanoparticle to protein ratio was calculated to gauge the purity of each preparation (Webber & Clayton, 2013) as a measure of quality control between preparations.

### 2.5 Time‐resolved fluorescence plate‐based detection of EV‐associated proteins

Time‐resolved fluorescence (TRF)‐based analysis of EV surface markers was performed as previously described (Webber et al., 2015). To assess ALIX and TSG101 expression, captured EVs were lysed with 100 μL 1X RIPA buffer on the plate prior to incubation with primary antibodies (Santa Cruz Biotechnology) and the remaining protocol was conducted previously (Webber et al., 2015).

### 2.6 SDS‐PAGE and western blot

Cells were lysed in lysis buffer (20 nM Tris, pH 7.5, 135 mM NaCl, 5% [v/v] glycerol, 50 nM NaF, 0.1% Triton X‐100), as described previously (Dunlop et al., 2014), with added protease inhibitors. Following sonication, centrifugation and protein quantification, samples were prepared in 4X LDS sample buffer (Invitrogen) with 25 mM dithothreitol (DTT). Lysates were separated by a 4%–12% gradient gel (Invitrogen), transferred onto PVDF membranes, blocked, then incubated with primary antibodies against TSC2 (catalogue #3990), total S6K1 (catalogue #9202), phospho‐S6K1 (T389) (catalogue #9205), pan‐Akt (catalogue #4691), phospho‐Akt (S473) (catalogue #4060) (all Cell Signaling Technology), ALIX (catalogue #sc‐166952), TSG101 (catalogue #sc‐7964), GRP94 (catalogue #sc‐393402) (all Santa Cruz), GAPDH (Novus Biologicals catalogue #1A10), overnight at 4°C. Following washing, secondary antibody incubation and further washes, proteins were visualised using LI‐COR ECL Reagent and a C‐DiGit® Blot Scanner (both LI‐COR Biotechnology) or Amersham ECL reagent and an ImageQuant 800 imaging system (both Cytiva).

### 2.7 Proteome profile antibody array

Confluent cells were treated with 0.7 μg/mL GolgiStop™ and 1 μg/mL GolgiPlug™ Protein Transport Inhibitors (Thermo Fisher Scientific) for 18 h to prevent cytokine release. Cells were lysed with 1X radioimmunoprecipitation assay (RIPA) buffer with phenylmethylsulfonyl fluoride, sodium orthovanadate and kit inhibitor cocktail (Bio‐Rad Laboratories), prior to protein quantification. Separately, EVs were isolated as described above. Total of 180 μg protein per cell line or EV preparation was incubated with the Proteome Profiler Human XL Oncology Array (R&D Systems) and processed as per the manufacturer's instructions. Membranes were imaged by chemiluminescence using C‐DiGit® Blot Scanner (LI‐COR) and analysed by densitometry using ImageJ software (version 1.50i).

### 2.8 Enzyme‐linked immunosorbant assay (ELISA)

Candidate protein expression was assessed using commercially available ELISA kits (DuoSet ELISA Development System, R&D Systems) as per manufacturer's instructions.

### 2.9 Functional enrichment analysis

FunRich: Functional Enrichment Analysis Tool software (Fonseka et al., 2021; Pathan et al., 2015) was used to conduct analysis. Genes encoding proteins (Table S1) with elevated expression in *TSC2*‐deficient cells and EVs were analysed against pre‐installed datasets on FunRich, namely Gene Ontology database, Human Protein Reference Database (Keshava Prasad et al., 2009), Entrez Gene (Maglott et al., 2007) and UniProt (UniProt Consortium, 2010). Categories with less than three gene hits were excluded and remaining GO terms with greatest fold enrichment scores were determined.

### 2.10 Rapamycin treatment assays

Total 10 ng/mL rapamycin in fresh media was added to confluent cells for 4 days. Cell‐conditioned media was collected, cells and debris removed by centrifugation and 0.22 μm filtration. Particles/mL secreted from each sample was assessed by NTA.

### 2.11 Cell viability assays

Fifty thousand fibroblast cells were plated in low‐serum Fibroblast Growth Medium 2 and allowed to adhere overnight. Cells were then washed twice in PBS before incubation in serum‐free DMEM with or without 100 μg/mL EV treatment. After 72 h, cell were trypsinised and cell viability and number were assessed using a NucleoCounter NC‐3000 (Chemometec, Denmark).

### 2.12 Growth factor secretion assays

Confluent fibroblasts were serum‐starved for 24 h, then treated with 200 μg/mL EVs suspended in serum‐free media for 4 days. Cell‐conditioned media was collected and subjected to ultracentrifugation at 100,000*g* for 2 h to deplete EVs. Resulting cell‐conditioned media was probed for VEGF and HGF expression using ELISAs as described above.

### 2.13 Isolation and detection of EVs from patient plasma

Plasma samples from TSC patients with a *TSC2* mutation were obtained from the TSC Alliance Biosample Repository (Van Andel Institute, Michigan, USA). Age and sex matched healthy donor plasma samples were obtained from the Cardiff University Biobank (University Hospital of Wales, Application Number 21‐0002). Plasma EVs were isolated using Exo‐spin™ midi columns (CELL Guidance Systems), and assessed for CD9 and CD63 expression using TRIFic™ detection assays (CELL Guidance Systems), using established protocols similar to as published (Welton et al., 2015).

### 2.14 Combined EV biomarker analysis

Combined biomarker scoring was performed based on Song et al. (2020). Briefly, receiver operating characteristic (ROC) curves of each biomarker were used to determine the optimal cutoff value based on the Youden index. Each biomarker was dichotomised, such that values above the cutoff scored 1 point and those below the cutoff value scored zero points. The overall score was the sum of the individual biomarker scores for endoglin, VEGF and enolase γ.

### 2.15 Statistics

All experiments were replicated at least three times with similar results. Graphs were prepared using GraphPad Prism, version 8 (GraphPad Software), which was also used to conduct statistical analyses. The data are presented as the mean ± SD. For experiments consisting of two experimental groups, groups were compared using a two‐tailed Mann Whitney non‐parametric test. To analyse three or more groups for statistical significance, one‐way ANOVA with Tukey's multiple‐comparison post hoc test was used, or Kruskal‐Wallis ANOVA with Mann‐Whitney post‐hoc tests for non‐normally distributed groups. A *p* value of less than 0.05 was deemed to be statistically significant. *p* values are marked by asterisks as follows: *p < 0.05; **p < 0.01; ***p < 0.001 and ****p < 0.0001; ns = non‐significant.

### 2.16 Study approval

Analysis of human samples used in this study was approved by Cardiff University School of Medicine Research Ethics Committee (SMREC reference: 19.84). Healthy donor samples were obtained from Cardiff University Biobank (REC No 18/WA/0089). TSC patient samples were obtained from TSC Alliance (IRB Study Number 15039‐05). Written informed consent was received prior to participation.

## 3 RESULTS

### 3.1 EVs isolated from TSC cells are of characteristic size, and express tetraspanins and ESCRT‐associated proteins

*TSC2*‐expressing (*TSC2+*) and ‐deficient (*TSC2*‐) cells derived from a patient angiomyolipoma were characterised based on expression of known cellular markers. The presence of Vimentin, but absence of both Desmin and Cytokeratin 18 confirmed the mesenchymal origin of these cells (Figure S1a). We also assessed expression and localisation of tetraspanins (CD9, CD63 and CD81) and Lysosome‐Associated Membrane Protein 2 (LAMP2), which is often cited for its role in EV biogenesis (Kowal et al., 2016; O'Brien et al., 2020). When comparing *TSC2*+ and *TSC2*‐ cells, we observed little difference between CD9 and CD63, but localisation of CD81 appeared to be altered in *TSC2*‐ cells, where CD81 appeared to be preferentially localised to the periphery of the cell (Figure S1b). Furthermore, enhanced peripheral localisation of LAMP2 in *TSC2*‐ cells (Figure S1b) supports the theory that endosomal functions are likely altered in the *TSC2*‐ cells. Altered CD81 and LAMP2 in *TSC2*‐ cells could potentially result in an altered EV secretion. This led us to assess EVs secreted by these cells.

**FIGURE 1:**
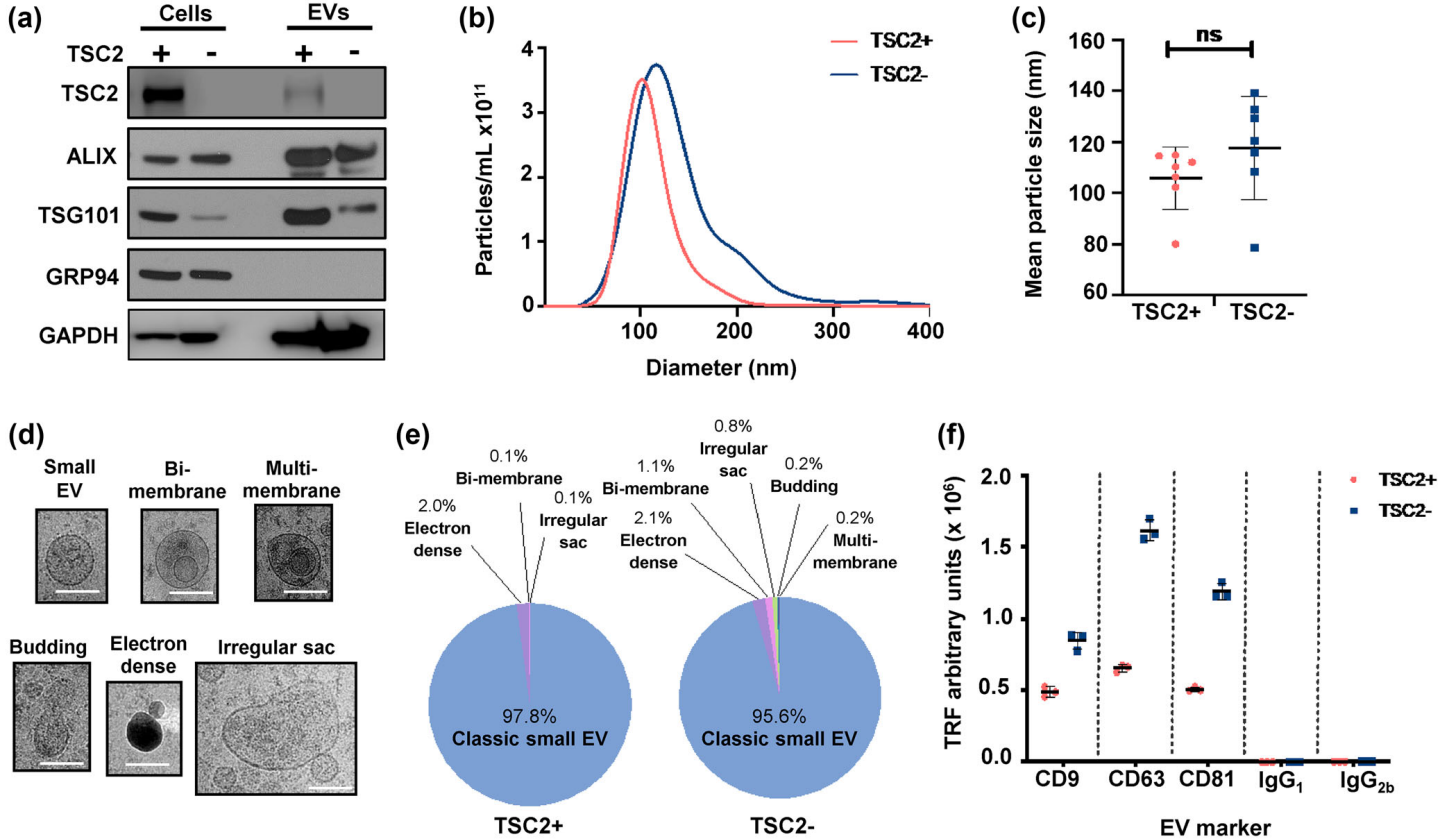
Isolated EVs were characterised using multiple complimentary techniques to determine EV molecular characteristics, size distribution and morphology. Expression of TSC2 and ESCRT‐associated proteins in cell and EVs lysates was assessed by western blot (10 μg protein/well) (a). Size distribution profiles of secreted vesicles (b) and mean sizes (*n* = 7 per cell line) (c) were assessed by NTA. Graphs plotted show mean ± standard deviation (SD). Statistical analysis was conducted using two‐tailed unpaired Mann Whitney non‐parametric test, ns = non‐significant. Vesicle morphology was assessed by cryo‐EM, scale bar = 150 nm (d) and categorised based on their morphology (e). Tetraspanin CD9, CD63 and CD81 expression on EV surfaces was examined using a TRF‐based plate assay (0.5 μg/well), technical triplicates shown (f). Blots and graphs shown are representative of three independent experiments. Cryo‐EM and subsequent analysis was from one experiment; 33 fields of view for TSC2+ sample, 46 fields of view analysed for TSC2‐ sample. Scale bar = 150 nm.

*TSC2*+ and *TSC2*‐ cells grown in high‐density cultures were confirmed to maintain the same signalling and response to rapamycin as seen in standard cell culture (Figure S2). Extracellular vesicles secreted from *TSC2*+ and *TSC2*‐ cells were isolated and subjected to rigorous characterisation. First, we compared whole cell lysates with EV lysates by western blot. Upon matched loading of cell and EV lysates, based on input protein, EVs showed enrichment of endolysosomal‐associated proteins: ALIX and TSG101 (Figure 1a). Interestingly, whilst levels of ALIX remained consistent when comparing either both cell types or their EVs, levels of TSG101 appeared to be reduced in both cell and EV lysates from *TSC2*‐ cells. Both ALIX and TSG101 were enriched within EV lysates compared to the respective cell lysates. In contrast, the endoplasmic reticulum (ER)‐associated protein GRP94 was enriched within cell lysates compared to EV lysates and detected at similar levels in *TSC2*+ and *TSC2*‐ cell lysates (Figure 1a). Additionally, we observed that TSC2 was detectable in EVs derived from *TSC2*+ cells (Figure 1a), which we believe to be a novel finding. Whilst GAPDH was detectable in all samples, it appeared to be enriched within EV lysates, compared to cell lysates, as has been reported previously (Shephard et al., 2021). Vesicle size was assessed by nanoparticle tracking analysis (Figure 1b), which showed that the majority of vesicles ranged from 30 to 150 nm in diameter. The mean diameter was not statistically significantly different between *TSC2+* and *TSC2‐* derived EVs (Figure 1c). Next, cryo‐EM was used to visualise these isolated EVs and categorise them based on morphology (Figure 1d,e). Cryo‐EM images showed that the majority of secreted vesicles were of small size, in the range of 20–150 nm in diameter (*TSC2*+ = 97.8%; *TSC*2‐ = 95.6%). Other bi‐membrane vesicles (*TSC2*+ = 0.1%; *TSC2*‐ = 1.1%) and larger irregular‐shaped sacs (*TSC2*+ = 0.1%; *TSC2*‐ = 0.08%) were also observed (Figure 1d), but in much fewer numbers (Figure 1e). Other budding vesicle morphologies and multi‐membrane vesicles were detected in *TSC*2‐ isolates, both at very low levels (both 0.2%). Next, we assessed the expression of the tetraspanins CD9, CD63 and CD81 on EV surfaces. Both *TSC2+* and *TSC2‐* EVs were positive for CD9, CD63 and CD81 (Figure 1f). Expression of all three tetraspanins appeared elevated on TSC2‐ EVs when compared to an equal amount of *TSC2+* EVs. These data highlight the successful isolation of both *TSC2*‐ cell‐derived and *TSC2*+ cell‐derived EVs, characterised based on guidance from the *International Society of Extracellular Vesicles* pertaining to the Minimal Information for Studies of Extracellular Vesicles (MISEV) (Lötvall et al., 2014; Théry et al., 2018).

### 3.2 TSC2‐ EVs have a distinct protein cargo

**FIGURE 2:**
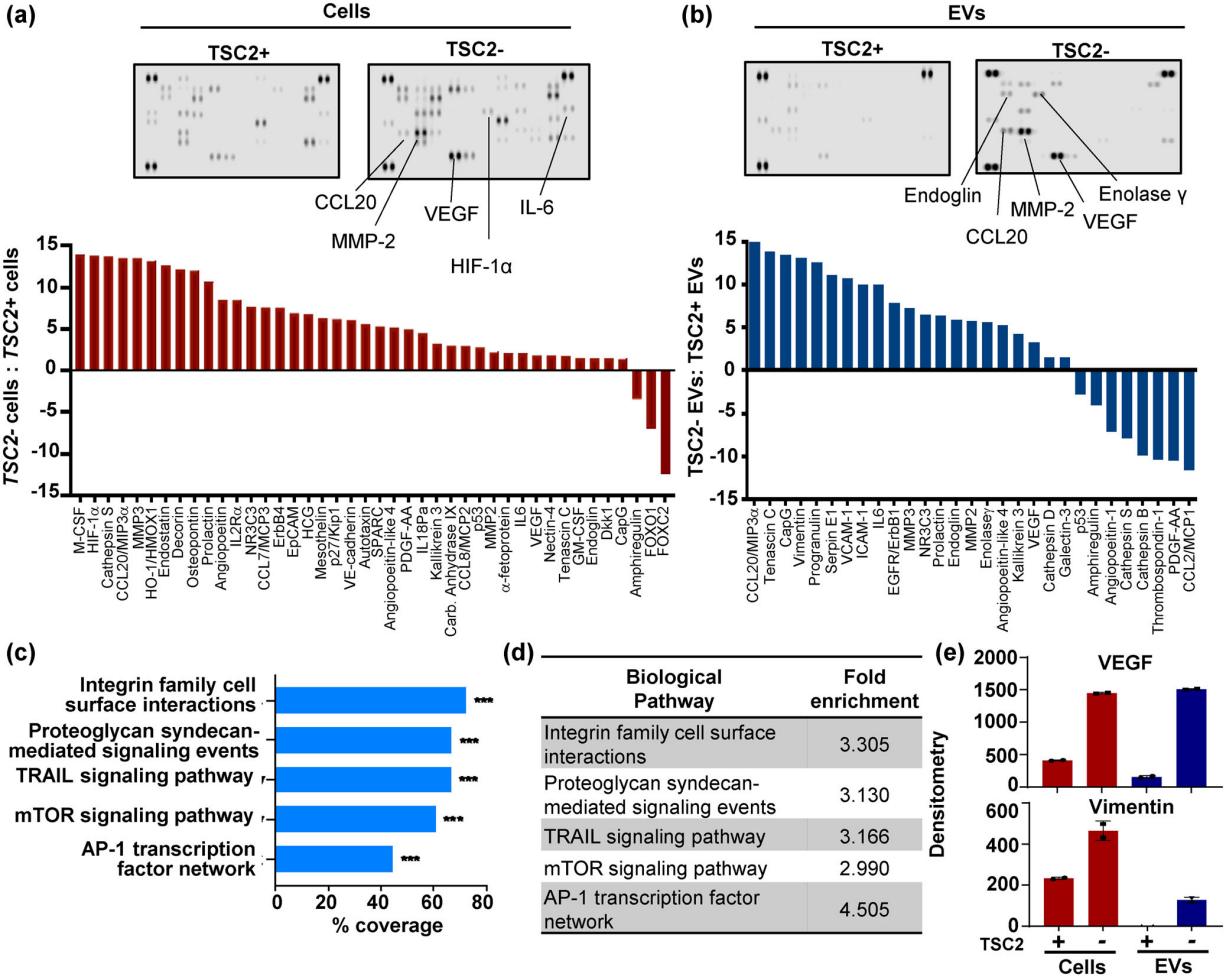
Proteome profiling of *TSC2+* and *TSC2‐* cells and cell‐derived EVs. Whole cell lysates (180 μg protein) and EVs (180 μg protein) derived from *TSC2+* and *TSC2–* cells (a), and EVs (b) were assessed by Proteome Profiler Human XL Oncology Array (R&D Systems) followed by densitometric analysis. Waterfall plots show fold increases/decreases of >2‐fold (*n* = 1) (a,b). Upregulated proteins in *TSC2‐* EVs were subject to functional enrichment analysis, and biological pathways with the greatest coverage (%) were ranked in descending order (c). Hypergeometric *p* values shown, where *** = *p* < 0.001. Fold enrichment in top‐ranking biological pathways shown in (d). Expression of two eIF4F‐regulated proteins (VEGF and Vimentin) is shown in (e).

We hypothesised that EV cargo may be functionally important to TSC pathology. We profiled the protein cargo by expression analysis of 84 tumour‐associated proteins in *TSC2+* and *TSC2‐* cells (Figure 2a) and their cell‐derived EVs (Figure 2b) using the Proteome Profiler Human XL Oncology Array (R&D Systems). We found altered expression of 42 tumour‐associated proteins in *TSC2*‐ cells relative to *TSC2*+ cells (Figure 2a). Some of the proteins with elevated expression in *TSC2*‐ cells have previously been linked to mTORC1‐driven tumours in TSC, including HIF‐1α, MMPs, VE‐cadherin, IL‐6 and VEGF (Bertolini et al., 2018; Dodd et al., 2015; Lee et al., 2010; Shu et al., 2010; Wang et al., 2021; Young et al., 2013). 29 tumour‐associated proteins were found to have altered expression in EVs from *TSC2*‐ cells compared to the EVs from *TSC2*+ controls. Of these, 21 proteins were enriched in *TSC2‐* EVs, with IL‐6, MMPs, VEGF, Galectin‐3 previously associated with mTORC1‐driven tumours (Dodd et al., 2015; Klover et al., 2017; Lee et al., 2010; Shu et al., 2010; Young et al., 2013). Eight proteins were observed at lower levels in *TSC2‐* EVs, than *TSC2+* EVs (Figure 2b). In the context of both the cell lysates and EV lysates, the majority of identified protein changes have not been previously associated with TSC pathology.

To elucidate likely functional properties of the EVs based on their protein cargo, we conducted functional enrichment gene ontology analysis on *TSC2*+ and *TSC2*‐ cells and their EVs. We focussed on genes encoding the observed upregulated proteins (as in Figure 2a,b), which were analysed for enriched gene networks under the gene ontology term ‘Biological pathways’, using functional enrichment gene ontology software FunRich (Fonseka et al., 2021; Pathan et al., 2015). Gene names corresponding to upregulated proteins are annotated in Table S1. We identified enriched biological pathways in proteins upregulated in *TSC2*‐ cells compared to *TSC2+* cells (Figure S3a); and in *TSC2‐* EVs, compared to their respective *TSC2*+ EVs (Figure 2c,d). In proteins upregulated in *TSC2‐* EVs (compared to *TSC2*+ EVs), the two top‐ranking biological pathways significantly enriched were *Integrin family cell surface interactions* (3.305 fold enrichment) and *proteoglycan‐syndecan mediated signalling events* (3.130 fold enrichment), which was not surprising given their known roles in EV effects on migration and adhesion (Hurwitz & Meckes, 2019) and EV biogenesis and uptake (Cerezo‐Magaña et al., 2020). The next biological pathways most significantly enriched were the *TRAIL signalling pathway*, involved in apoptosis cascades (3.166‐fold enrichment), and the *mTOR signalling pathway*, the known intracellular driver of TSC tumours (2.990‐fold enrichment). Both of these biological pathways had greater fold enrichments in *TSC2*‐ EVs compared to that found in *TSC2*‐ cells, which could suggest their implication in disease in the extracellular space (Figure S3a). Consistent with the gene ontology analysis, VEGF and vimentin which are sensitive to mTORC1‐eIF4F‐regulated mRNA translation, were upregulated in *TSC2*‐ cells and EVs (Figure 2e). *AP‐1 transcription factor network*, with known roles in signalling associated with proliferation, angiogenesis and survival, in various cancers (Wu et al., 2021), was also a high‐ranking biological pathway found in TSC2‐ EVs (4.505‐fold enrichment). Biological pathways linked to proteins upregulated in both *TSC2‐* cells and *TSC2‐* EVs are shown in Figure S3b.

A comparison of proteins present in *TSC2‐* EVs compared to *TSC2‐* cell lysates revealed distinct expression patterns. Of the proteins which had higher expression in *TSC2–* EVs compared to *TSC2+* EVs, the majority of these proteins were more readily detected in the *TSC2–* cells than in their EVs. Several proteins (MMP2, VEGF, EGFR/ErbB1, Endoglin and Galectin 3) had similar abundance in both *TSC2–* EVs and *TSC2‐* cell lysates. ICAM‐1, CCL20 and VCAM1 however were more readily detectable in *TSC2‐* EVs compared to *TSC2–* cells (Figure S3c) suggesting these proteins may be preferentially loaded into EVs.

### 3.3 Validation of selected protein expression in TSC2+ and TSC2‐ EVs

**FIGURE 3:**
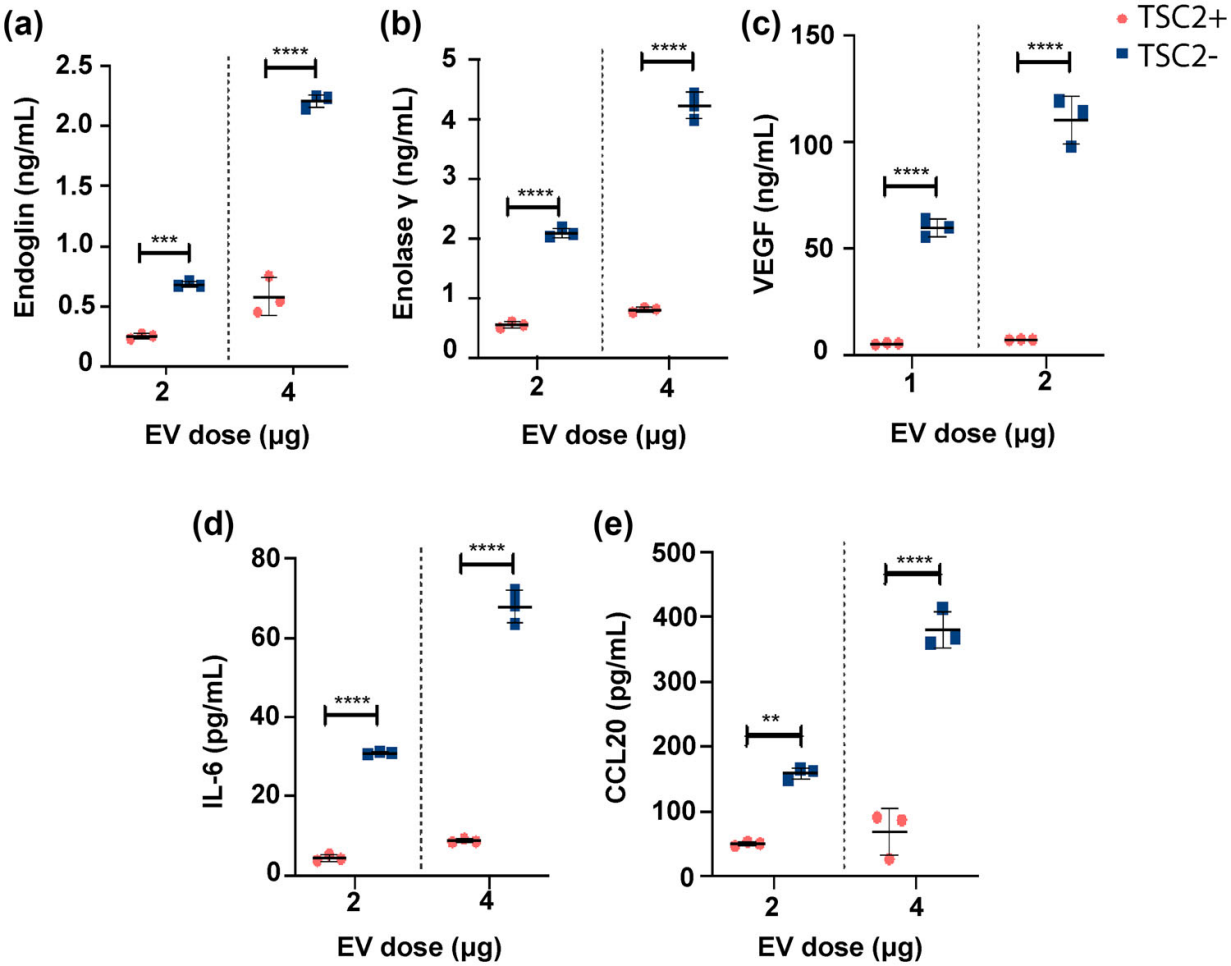
*TSC2‐* EVs are enriched for selected proteins involved in tumour biology. EV expression of selected protein candidates, identified by proteome profiler array, were validated by ELISA‐based assessment of two doses of EVs (as stated per graph), lysed using RIPA buffer. Graphs plotted show technical triplicates, bars denote mean ± SD. Statistical analysis conducted using one‐way ANOVA with Tukey's multiple comparisons test. **p < 0.01; ***p < 0.001; ****p < 0.0001 (a–e).

To investigate proteins of potential functional importance in TSC, we combined our list of novel TSC‐associated proteins with our gene ontology analysis and chose to focus on proteins that were strongly upregulated (Figure 2b) and likely to have a functional consequence in TSC, based on their reported cellular functions. From this, we validated that endoglin, enolase γ, VEGF, IL‐6 and CCL20 protein levels were significantly elevated in *TSC2‐* EVs, compared to *TSC2*+ EV controls (Figure 3a‐e).

### 3.4 Rapamycin affects EV secretion and cargo

**FIGURE 4:**
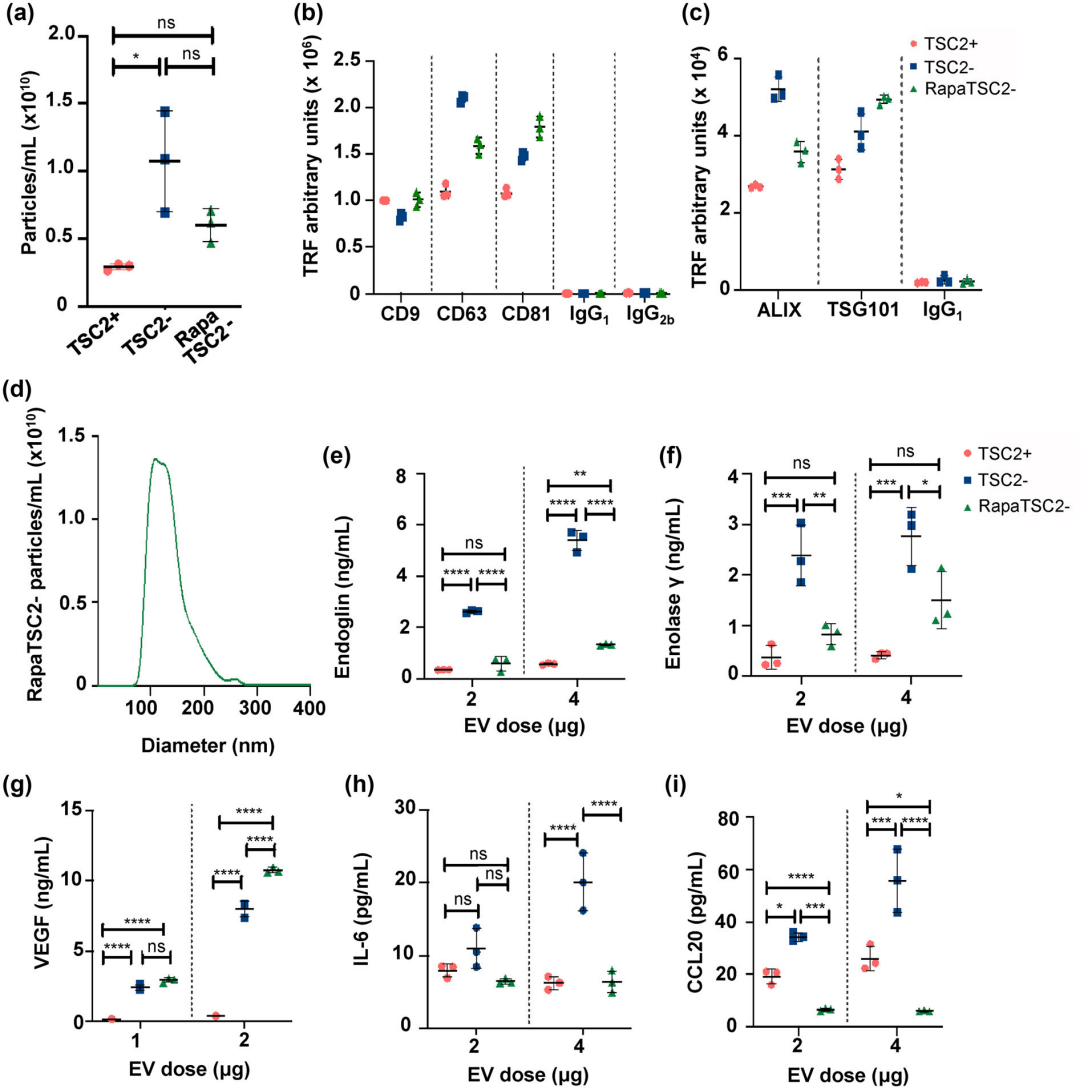
Rapamycin treatment reduces EV secretion and cargo loading of selected protein targets, compared to untreated cells. EV secretion from *TSC2+*, *TSC2–* and rapamycin‐treated *TSC2–* (rapa*TSC2–*) cells was assessed by NTA (a); expression of tetraspanins on the EV surface (b) and ESCRT‐associated proteins within lysed EVs (c) was assessed by TRF‐based plate assay; vesicle size distribution was assessed by NTA (d). Expression of protein cargo targets was assessed by ELISA (e–i). Representative graphs of three independent experiments for each are shown. Graphs plotted show biological triplicates in (a) and technical triplicates in (b, c, e–i). Bars show mean ± SD. Statistical analysis conducted using one‐way ANOVA with Tukey's multiple comparison's test. ns = non‐significant; *p adj. < 0.05; **p < 0.01; ***p < 0.001; ****p < 0.0001.

Rapamycin is a well‐characterised inhibitor of mTORC1 signalling. Its derivatives, rapalogs, are clinically approved to treat TSC tumours as well as some sporadic cancers (Hua et al., 2019; Ní Bhaoighill & Dunlop, 2019). Analysis of rapamycin's mechanism of action is mostly focussed on its impact on intracellular signalling, with limited knowledge of how it may influence intercellular signalling or the tumour microenvironment. Therefore, we investigated the effect of rapamycin on EV secretion and cargo. To achieve this, *TSC2*‐ cells were maintained in long‐term culture with media supplemented with 10 ng/mL rapamycin, and resultant EVs compared to those from untreated *TSC*2‐ cells. The rapamycin dose was chosen to reflect the trough levels of TSC patients across multiple clinical trials (Bissler et al., 2013, 2008; Davies et al., 2011) to emulate the scenario in the patient population who tend to be treated long‐term with rapamycin to control their tumours. Nanoparticle tracking analysis revealed that conditioned media from *TSC2‐* cells contained more particles than media from *TSC2+* cells, suggesting that *TSC2‐* cells secrete more EVs. We found that long‐term rapamycin‐treatment of *TSC2*‐ cells resulted in a potential reduction in EV secretion compared to untreated *TSC2*‐ cells (Figure 4a). Despite a consistent trend being observed it did not reach statistical significance due to the variability in EV quantitation. However, this result is consistent with that seen in WT and *Tsc2* mutant murine renal cell lines, where rapamycin significantly reduced EV production (Kumar et al., 2021). EVs from rapamycin treated *TSC2*‐ cells expressed the tetraspanins CD9, CD63 and CD81 as well as luminal ALIX and TSG101 at similar levels to *TSC2*+ and *TSC2‐* EVs (Figure 4b,c). Importantly, a decrease in both CD63 and ALIX detection in EVs from rapamycin‐treated *TSC2*‐ cells compared to EVs from untreated cells points to an altered EV morphology in response to rapamycin. NTA analysis also revealed that rapamycin did not affect the size distribution profile of vesicles (Figure 4d). To further explore whether EV cargo was modulated by rapamycin treatment, we assayed levels of our protein candidates increased in *TSC2‐* EVs; namely endoglin, enolase γ, VEGF, IL‐6 and CCL20. We found that rapamycin significantly reduced levels of EV‐associated endoglin, enolase γ, IL‐6 and CCL20 (Figure 4e,f,h,i) compared to untreated *TSC2‐* EVs, bringing candidate protein expression levels more in line with those observed in *TSC2+* EVs. This could potentially indicate a novel therapeutic function of rapamycin by reducing tumour‐supporting cargo loading into EVs. Interestingly, in the case of VEGF, rapamycin treatment did not reduce its protein abundance in *TSC2‐* EVs in comparison to untreated cells, and there were indications VEGF cargo in EVs from rapamycin‐treated cells was significantly increased (Figure 4g). This is in line with previous reports showing that high VEGF protein levels are not attenuated by rapamycin in TSC models due to TSC2 regulation of VEGF through mTOR‐independent pathways (Brugarolas et al., 2003).

### 3.5 TSC2‐ EVs enhance cell viability of recipient fibroblasts and induce the secretion of VEGF and HGF

**FIGURE 5:**
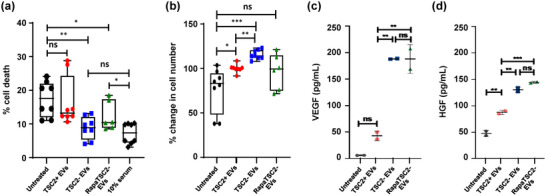
*TSC2*‐ cell‐derived EVs alter viability, proliferation and growth factor secretion of recipient fibroblasts. Recipient fibroblast cells were starved for 72 h with or without EV treatment as indicated. Cell viability (a) and cell number (b) were assessed and are plotted as box and whisker plots, minimum to maximum, *n* ≥ 3. Statistics were conducted using Kruskal‐Wallis one‐way ANOVA and Mann‐Whitney post‐hoc tests. Cell‐conditioned media from EV‐stimulated fibroblasts was subjected to ultracentrifugation to pellet and deplete EVs, and remaining media was probed for VEGF and HGF expression by ELISA, loaded by total protein (1 μg) (c,d). Representative example shown of three independent experiments for each. Graphs plot technical duplicates; error bars denote mean ± SD. Statistical analysis conducted using one‐way ANOVA with Tukey's multiple comparison's test. ns = non‐significant; *p < 0.05; **p < 0.01; ***p < 0.001.

To determine the ability of these EVs to influence their microenvironment we investigated whether they could alter the viability or cell number of recipient fibroblasts under starvation conditions. We found that EVs derived from *TSC2*‐ cells were as effective as serum at improving cell viability under starvation conditions, which was not seen for EVs from *TSC2*+ cells (Figure 5a). While EVs from rapamycin treated *TSC2*‐ cells also improved cell viability, they were not as effective as adding serum to the media, indicating some attenuation of potency of EVs from rapamycin treated *TSC2*‐ cells. EVs from *TSC2*‐ cells also induced more cell proliferation of recipient fibroblasts than EVs from *TSC2*+ cells (Figure 5b). EVs from rapamycin treated *TSC2*‐ cells were much more variable in their impact on cell number.

We then examined whether EV treatment could promote growth factor secretion from the recipient fibroblast cells. We analysed VEGF, due to the highly angiogenic nature of AML tumours, and HGF, due to its reported link to a cancer‐associated fibroblast state (Webber et al., 2015). Following the incubation period, all EVs were removed from the media by high‐speed centrifugation, so that only soluble, secreted factors were analysed. *TSC2‐* EVs were found to enhance VEGF and HGF secretion from recipient fibroblasts (Figure 5c,d), which we have previously shown to be a feature of a tumour‐supporting fibroblast phenotype (Webber et al., 2015). *TSC2*‐ EVs enhanced VEGF (*p* = 0.0019) and HGF (*p* = 0.0022) to a greater capacity than EVs from *TSC2*+ cells. EV‐induced secretion of VEGF and HGF was however not attenuated when *TSC2*‐ cells were treated with rapamycin (Figure 5c,d), suggesting *TSC2*‐dependent, but mTORC1‐independent regulation, as previously indicated (Brugarolas et al., 2003; Dodd et al., 2015).

### 3.6 EV‐associated cargoes are detectable in TSC patient plasma

**FIGURE 6:**
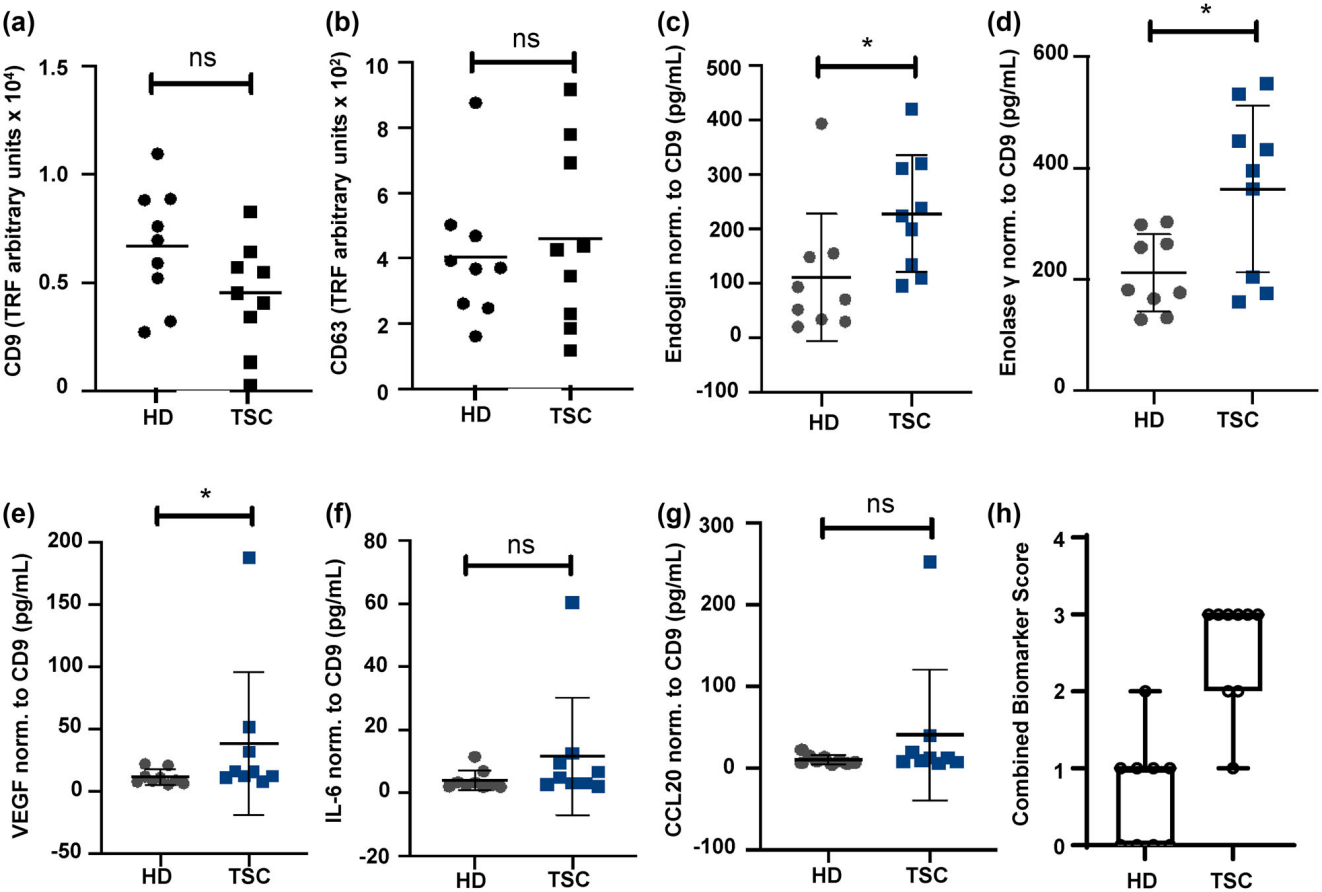
EVs were isolated from plasma samples by SEC and characterised prior to assessment of candidate protein cargo expression. Confirmation of EV‐rich fractions in all plasma samples (*N* = 18) by analysing CD9 and CD63 expression using TRIFic™ detection assays (CELL guidance systems) (a,b). Expression of protein cargo candidates in lysed EVs was assessed by ELISA, and normalised to CD9 expression (c‐g). A combined biomarker score was calculated and plotted as a box and whisker plot, minimum to maximum (h). Graphs plotted in (a–g) show mean ± SD. Statistical analysis conducted using unpaired two‐tailed Mann Whitney non‐parametric tests. ns = non‐significant; *p < 0.05.

To determine whether any of the EV cargo changes could translate to potential biomarkers of TSC, we isolated EVs from plasma samples from patients with a *TSC2* mutation and age and sex‐matched unaffected healthy donors (*N* = 9 patients/group; patient information tabulated in Table S2) using an established size exclusion chromatography‐based approach (Welton et al., 2015). We confirmed enrichment of CD9 and CD63 in size‐exclusion chromatography fractions 8–14 from both healthy donor and TSC patient samples (Figure 6a,b). We then analysed detection of our candidate proteins, normalised to CD9 levels, to investigate their potential application as disease biomarkers. We found significantly enriched expression of endoglin (*p* = 0.0188), enolase γ (*p* = 0.0400) and VEGF (*p* = 0.0400) in EVs from TSC patient plasma EVs compared to EVs from unaffected healthy donor plasma (Figure 6c‐e), which matches our in vitro findings (Figure 3a‐c). IL‐6 (*p* = 0.1359) and CCL20 (*p* = 0.2973) expression was not significantly altered in TSC patient plasma EVs compared to unaffected healthy donor EVs (Figure 6f,g). As there was overlap in expression between the healthy donor and TSC groups, we investigated whether a combined score may discriminate between the groups more effectively. We set cut‐off values for endoglin, enolase γ and VEGF based on the Youden index (Akobeng, 2007) and assigned each patient a score of 1 for each of the three biomarkers if their reading was above the cut‐off value. Scores of ≤1 identified 8/9 healthy donors, while scores of ≥2 identified 8/9 TSC patients (Figure 6h). This data highlights the potential of EVs as a source of biomarkers to identify patients with TSC and could be used in future test development.

## 4 DISCUSSION

How TSC cysts prime their microenvironment to encourage and support growth is an emerging area. In the lung setting, TSC/LAM cells have been shown to induce senescence in neighbouring cells in a paracrine manner (Bernardelli et al., 2022). In a TSC renal cyst mouse model and TSC neural mouse model, wild‐type cells of the microenvironment were observed to take on an EV‐mediated *Tsc2*‐mutant disease phenotype (Kumar et al., 2021; Patel et al., 2016). Here, we provide further evidence that TSC cells can influence their microenvironment through EV‐mediated communication.

In the current study we show that mTORC1 activity does not affect EV size, in agreement with past studies (Gao et al., 2020; Zou et al., 2019). However, the AML mTORC1‐active cells examined here secreted more EVs than controls, as aligns with work in hepatic stellate cells (Gao et al., 2020) and murine kidney collecting duct cells (Zadjali et al., 2020). In contrast, EV secretion was reported to be increased following mTORC1 inactivation by serum or amino acid starvation *Tsc2*‐expressing MEF cells and C57BL/6 mice (Zou et al., 2019). The differences in these findings could be due to both the complex regulation of EV trafficking and secretion, as well as potential tissue‐specific regulation of mTORC1 (Wolff et al., 2017).

The EVs released by *TSC2*‐ cells have a different protein composition to EVs secreted from *TSC2*+ cells. Some of these proteins are already linked to TSC pathology, such as IL‐6 (Shu et al., 2010; Wang et al., 2021), MMPs (Broekaart et al., 2020; Lee et al., 2010), VEGF (Dodd et al., 2015; Young et al., 2013) and Galectin‐3 (Klover et al., 2017) although association of these proteins with TSC EVs is novel. We focussed on endoglin, enolase γ, VEGF, IL‐6 and CCL20 which were all enriched in *TSC2*‐ EVs, compared to *TSC2*+ EVs, due to known associations with tumour‐promoting functions. Endoglin is implicated in angiogenesis (Grange et al., 2011; Tian et al., 2018) while EV‐associated VEGF has been demonstrated to promote angiogenesis around tumours (Feng et al., 2018; Ko et al., 2019; Webber et al., 2015). Therefore, if these factors function similarly in TSC, they could work in concert to encourage growth of the dense angiogenic networks that characterise AMLs. Enolase γ facilitates accelerated glycolysis (Vizin & Kos, 2015) and so its delivery within the tumour microenvironment could help enhance cell growth. IL‐6 has recently been linked to TSC pathology, through a role in serine metabolism (Wang et al., 2021). Although cancer‐derived EVs have been shown to contain IL‐6 (Ramteke et al., 2015; Skog et al., 2009) the impact of EV‐delivered IL‐6 on cells within the tumour microenvironment has not been explored. Finally, CCL20 has not previously been linked to TSC, but it has been found on stromal cell EVs and implicated in migration and inflammation suppression (Mardpour et al., 2019). Therefore EV‐derived CCL20 could similarly influence the microenvironment in TSC.

To explore microenvironmental consequences, we looked at cell viability, proliferation and growth factor secretion in response to EV treatment. EVs from *TSC2*‐deficient cells were more effective than other EV types at improving cell viability and number under starvation conditions. EVs from *TSC2*‐deficient cells also induced secretion of significantly higher levels of VEGF and HGF than those EVs from control cells. These findings that TSC EVs can alter recipient cell biology support those previously observed in mouse models of TSC, where *Tsc1*‐deficient neuronal progenitors could alter the phenotype of neighbouring genetically wild‐type neuronal cells via delivery of exosomes in a mouse model (Patel et al., 2016) and EVs from *Tsc2*‐deficient murine inner medullary collecting duct cell lines could modify the transcriptome of recipient cortical collecting duct cells (Kumar et al., 2021). Furthermore, results from the current study are in agreement with our own previous studies which highlighted that cancer‐derived EVs can modulate the tumour microenvironment by inducing a fibroblast phenotype with pro‐angiogenic and tumour supporting function. Whilst elevation of αSMA expression is often regarded as a key marker of a cancer associated fibroblast (CAF) phenotype, our previous studies have shown this to be a poor indicator of a genuine tumour‐supporting phenotype. An elevated secretion of HGF was a better indicator of EV‐activated fibroblasts capable of enhanced tumour growth (Webber et al., 2015). Therefore, by improving cell viability and increasing cell proliferation, VEGF and HGF secretion from cells of the TME, our data suggests a more growth supporting environment is likely induced by *TSC2*‐ EVs than *TSC2*+ EVs.

Although rapalogs are clinically approved for manifestations of TSC and certain cancers (Gomes et al., 2022; Martelli et al., 2018; O'Shea et al., 2022; Palavra et al., 2017), their wider impact on the tumour microenvironment has not been extensively examined. We found a trend of reduced EV secretion from *TSC2*‐deficient cells following rapamycin treatment, similar to that previously reported in murine cells (Kumar et al., 2021), combined with altered protein packaging into the EVs in vitro. When normalised for protein content, EVs from rapamycin‐treated cells contained significantly reduced levels of endoglin, enolase γ, IL‐6 and CCL20 compared to untreated *TSC2*‐deficient cells, with levels comparable to that seen in EVs from control cells (Figure 4). The reduction in these pro‐angiogenic and pro‐tumoral factors could ameliorate the effects of *TSC2*‐ cells on their microenvironment, thus rapamycin may not only shrink TSC cyst and tumour size but also reduce the microenvironmental support for growth. In contrast, a proteomic screen of EVs from HeLa cells with and without rapamycin treatment found very few differences in cargo (Zou et al., 2019), which could be reflective of the different models used.

However, it is important to note that we observed that EVs from rapamycin‐treated *TSC2*‐ cells were just as effective as EVs from untreated *TSC2*‐ cells at inducing growth factor secretion from recipient fibroblasts. This indicates that not all features of *TSC2*‐ EVs are attenuated following treatment of EV secreting cells with rapamycin. Our finding that VEGF is still incorporated into EVs, and that VEGF secretion is induced in recipient cells despite rapamycin treatment, fits with previous findings where rapamycin was seen to only partially downregulate VEGF (Brugarolas et al., 2003). This further evidence of mTORC1‐independent links between *TSC2* loss and VEGF expression has potential implications for the effectiveness of rapamycin treatment, especially for highly angiogenic tumours.

To determine potential translational impact of these findings, we examined whether our EV‐associated candidate proteins were detectable within TSC patient plasma. The plasma EV signature will be reflective of the whole body, so in the case of TSC patients, these EVs will be derived from both TSC tumours (mTORC1‐active) and cells with a single *TSC2* mutation (normal mTORC1 levels). We successfully isolated EVs from healthy donors and TSC patients and were able to show that the changes observed in cell lines in vitro extended to the patient setting, despite the mixed cell sources of plasma‐derived EVs. Importantly, higher levels of EV‐associated endoglin, enolase γ and VEGF were detected in TSC patient plasma compared to healthy donors. When levels of these proteins were scored and combined, the majority of healthy donors and TSC patients were separated into two distinct groups. This indicates the potential utility of EVs as a source of biomarkers for identification of patients with TSC or for other mTORC1‐driven tumours.

## 5 CONCLUSIONS

Overall, this study is one of the first reported comparisons of the protein content of EVs derived from *TSC2*‐deficient, mTORC1‐active cells. Importantly, we show that the packaging of proteins linked to various tumour‐supporting signalling pathways is favoured in EVs derived from mTORC1‐active cells compared to those with normal mTORC1 activity. *TSC2*‐ EVs can induce cell proliferation and growth factor release from recipient fibroblasts. Our findings that rapamycin can attenuate some, but not all, EV functions could help explain the heterogenous response to rapalog treatment seen in TSC patients and our observations indicate that additional therapeutic targets for TSC may lie within intercellular signalling pathways and EV modulation. Whilst the use of EVs as TSC biomarkers requires further development, we have identified several EV‐associated proteins which when combined have the potential to differentiate patients with TSC from unaffected healthy donors.

## AUTHOR CONTRIBUTIONS

Muireann Ní Bhaoighill designed and performed research, analysed data and wrote the manuscript. Juan M. Falcón‐Pérez and Félix Royo performed cryo‐EM and reviewed the manuscript. Andrew R. Tee provided advice and helped with manuscript editing. Jason P. Webber designed and supervised the study, analysed data and wrote the manuscript. Elaine A. Dunlop designed and supervised the study, performed research, analysed data and wrote the manuscript.

## CONFLICT OF INTEREST STATEMENT

The authors report no conflict of interest.

## Supporting information

Supporting Information

Supporting Information

Supporting Information

Supporting Information

Supporting Information
