# Supplementary figures and images for: Tuberous Sclerosis Complex cell‐derived EVs have an altered protein cargo capable of regulating their microenvironment and have potential as disease biomarkers
Source: J Extracell Vesicles. 2023 Jun 19;12(6):12336. doi: 10.1002/jev2.12336 (PMC10279809; doi:10.1002/jev2.12336)

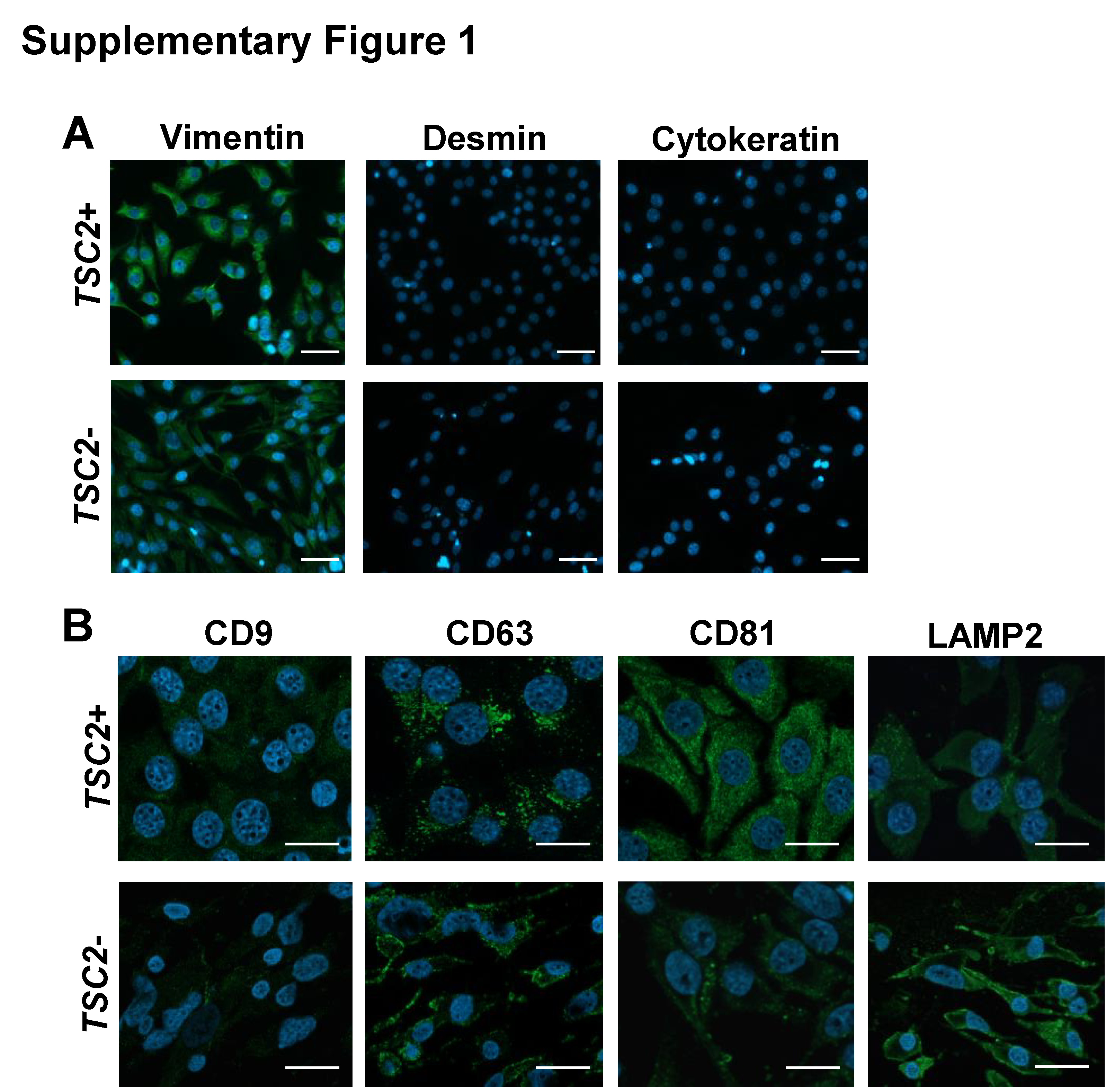

Supplement: Supplementary file 1 — Supporting Information [file JEV2-12-12336-s001.tif]

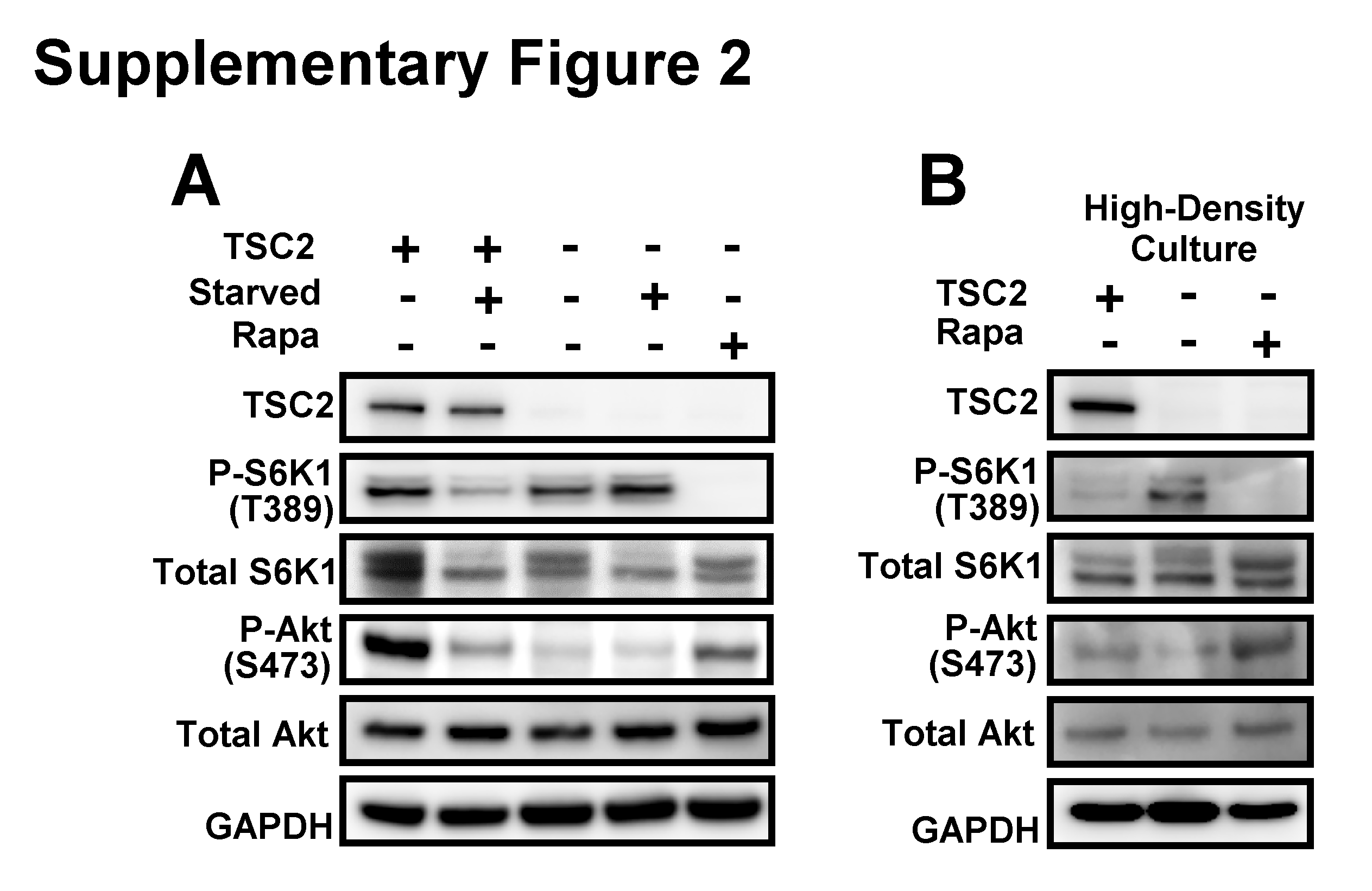

Supplement: Supplementary file 2 — Supporting Information [file JEV2-12-12336-s005.tif]

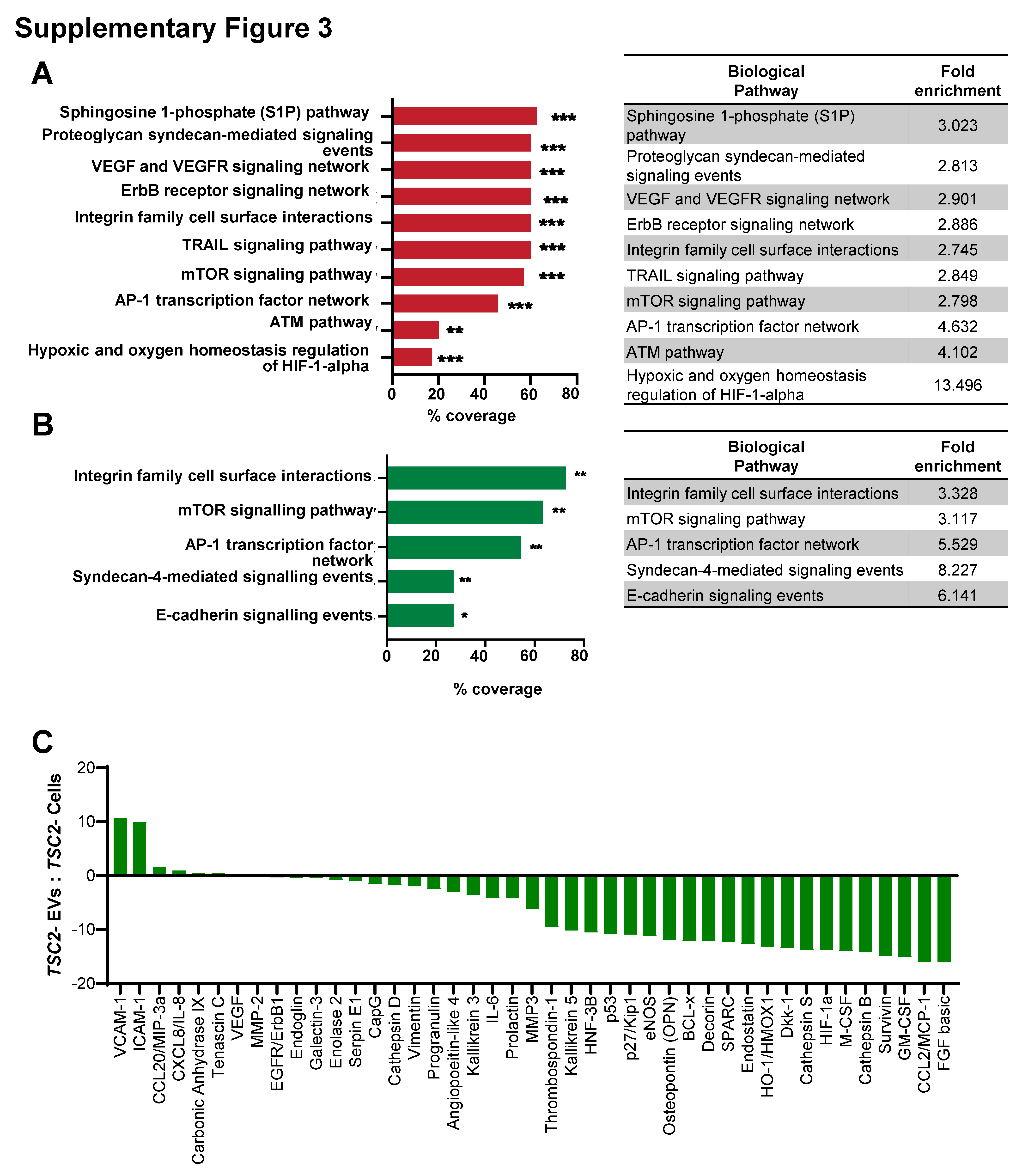

Supplement: Supplementary file 3 — Supporting Information [file JEV2-12-12336-s004.tif]

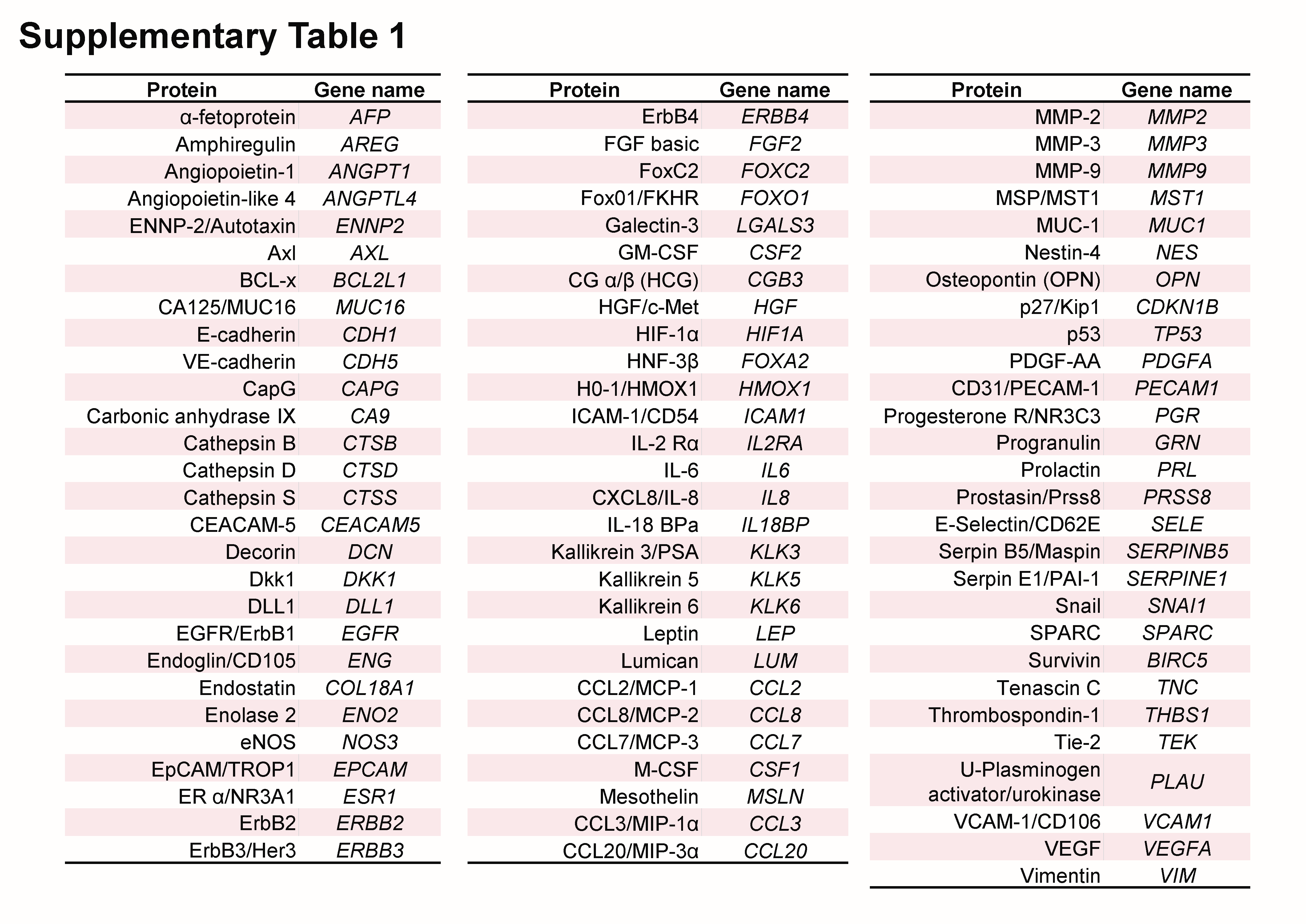

Supplement: Supplementary file 4 — Supporting Information [file JEV2-12-12336-s003.tif]

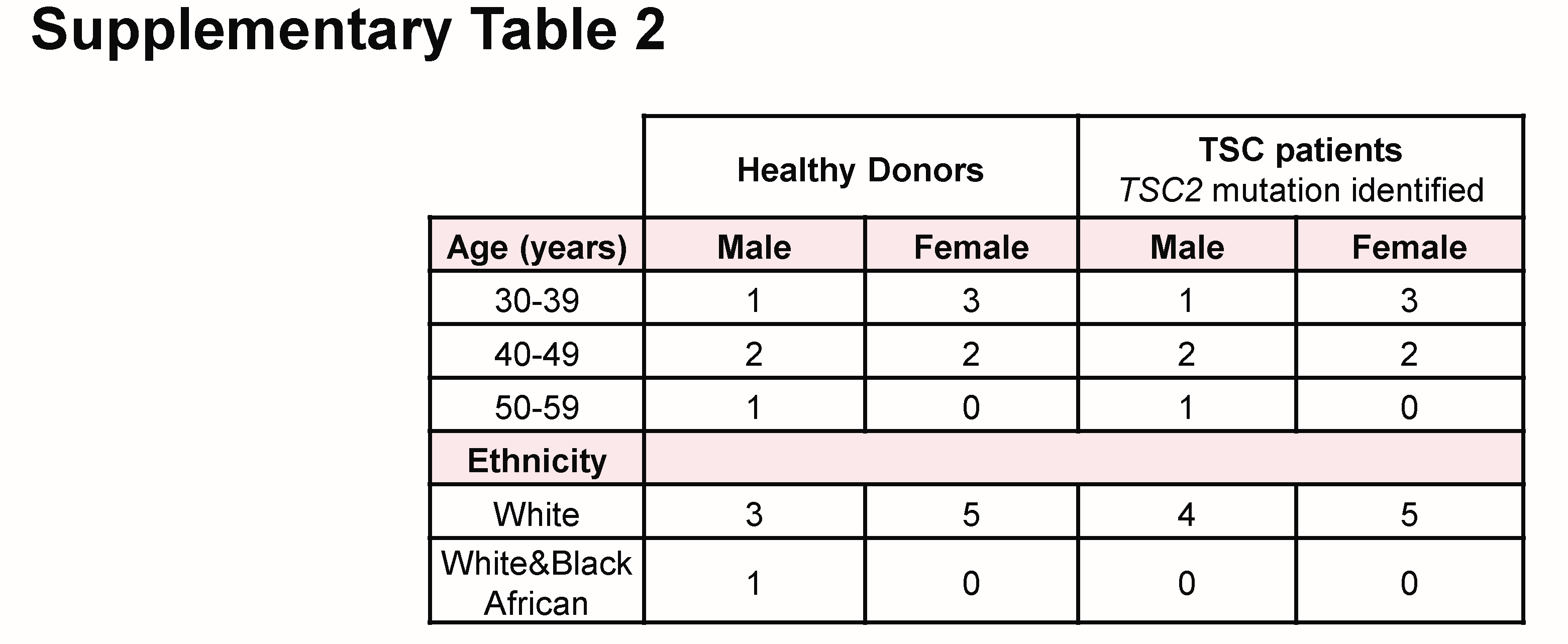

Supplement: Supplementary file 5 — Supporting Information [file JEV2-12-12336-s002.tif]
